# Author Correction: Task-evoked pupil responses reflect internal belief states

**DOI:** 10.1038/s41598-018-33559-9

**Published:** 2018-10-23

**Authors:** Olympia Colizoli, Jan Willem de Gee, Anne E. Urai, Tobias H. Donner

**Affiliations:** 1 Department of Neurophysiology and Pathophysiology, University Medical Center Hamburg-Eppendorf, Hamburg, Germany; 2 Department of Psychology, University of Amsterdam, Amsterdam, The Netherlands; 3 Amsterdam Brain & Cognition, University of Amsterdam, Amsterdam, The Netherlands

Correction to: *Scientific Reports* 10.1038/s41598-018-31985-3, published online 12 September 2018

In Figure 5b the p-value is incorrect. The correct Figure 5 appears below as Figure 1.

**Figure 1:**
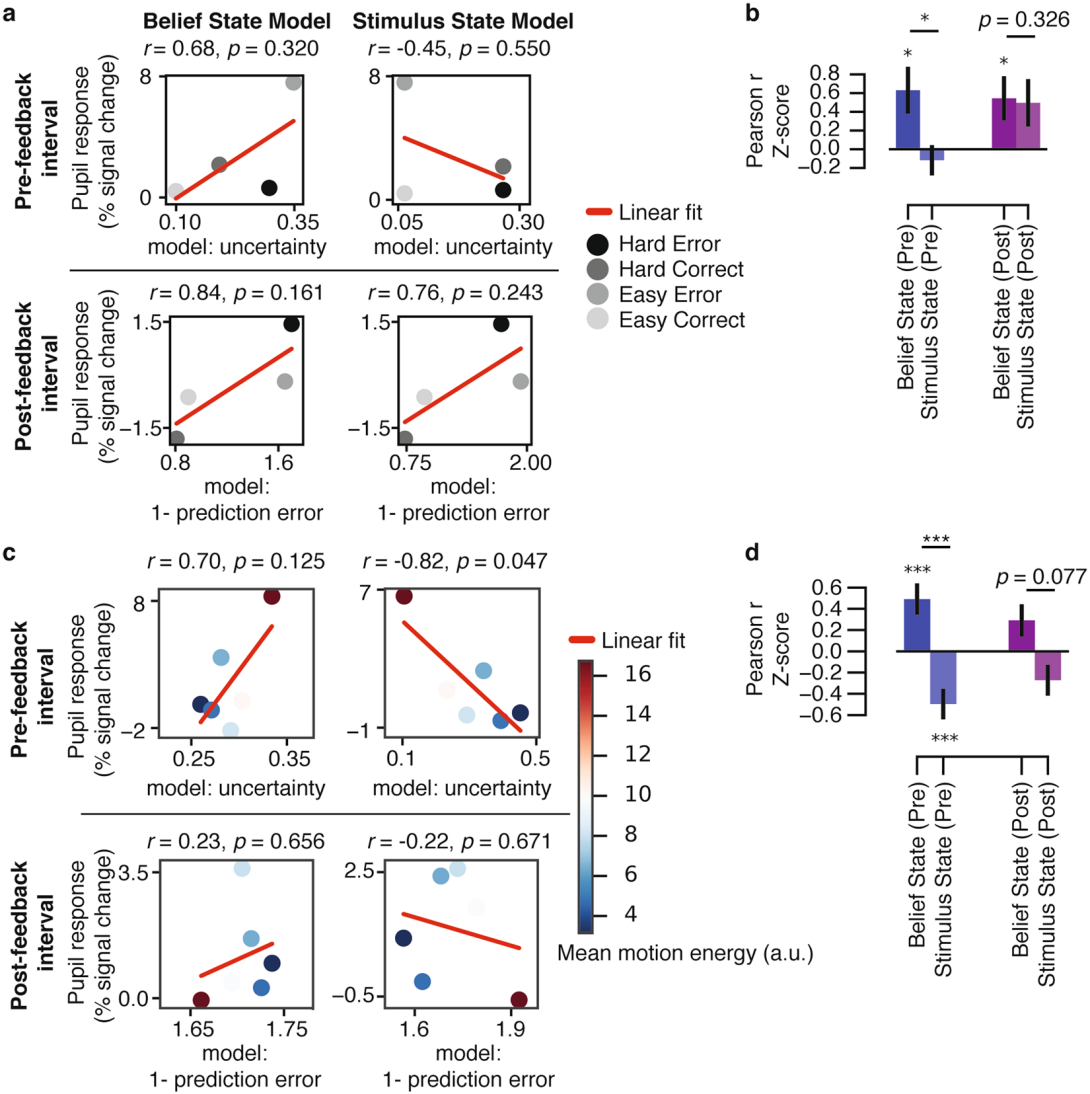
Model fits to pupil responses. (**a**) An example of the correlations (*r*) for a single subject. The four conditions of interest were defined by the Accuracy x Difficulty interaction. Easy and Hard conditions for the model parameters were averaged based on the coherence levels presented to each subject. (**b**) Group-level correlation coefficients (*r*) for the comparison of the model parameters and pupil responses, for the pre-feedback (Pre; −0.5–0 s) and post-feedback (Post; 3–6 s) intervals. (**c**) An example of the correlations for a single subject using model parameters simulated with motion energy (error trials only). Pupil responses were averaged within six equal-sized bins based on the model parameter for each interval. Evidence strength is represented by mean motion energy within each bin (color bar). (**d**) Group-level correlation coefficients (*r*) for the comparison of the model parameters (using motion energy) and pupil responses, for the pre-feedback (Pre; −0.5–0 s) and post-feedback (Post; 3–6 s) intervals (error trials only). Error bars, standard error of the mean (*N* = 15). **p < 0.01, ***p < 0.001.

As such, this Article contains an error in the Results section under subheading ‘Belief State Model predicts pupil responses quantitatively better than Stimulus State Model’,

“For the post-feedback interval, there was a trend towards a stronger correlation for the Belief State Model than the Stimulus State Model (p = 0.074).”

should read

“For the post-feedback interval, there was no difference between the models (p = 0.326)”.

