# Supplementary material for: Task-evoked pupil responses reflect internal belief states
Source: Sci Rep. 2018 Sep 12;8:13702. doi: 10.1038/s41598-018-31985-3 (PMC6135755; doi:10.1038/s41598-018-31985-3)
Supplement: Supplementary file 1 — Supplementary Information [file 41598_2018_31985_MOESM1_ESM.docx]

**Supplementary Information: Task-evoked pupil responses reflect internal belief states**

O. Colizoli^1,2^, J.W. de Gee^1,2^, A.E. Urai^1,2^, T.H. Donner^1,2,3,*^

^1^ Department of Neurophysiology and Pathophysiology, University Medical Center Hamburg-Eppendorf, Hamburg, Germany

^2^ Department of Psychology, University of Amsterdam, Amsterdam, The Netherlands

^3^ Amsterdam Brain & Cognition, University of Amsterdam, Amsterdam, The Netherlands


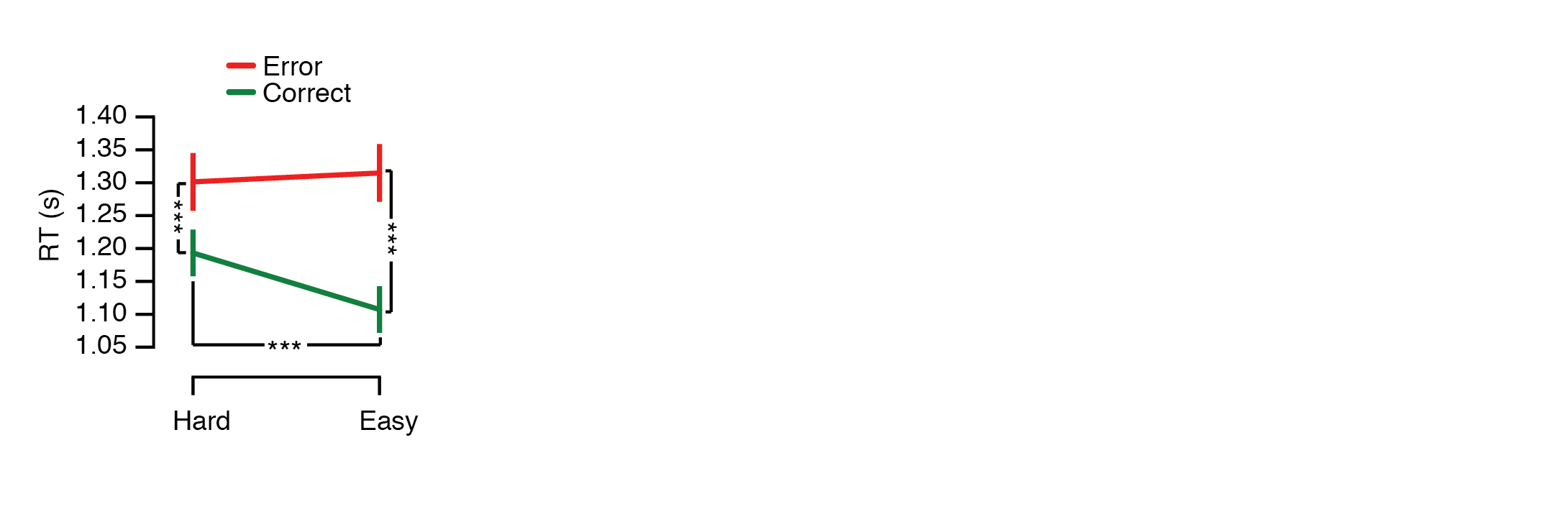


**Supplementary Figure S1. RT scales with decision uncertainty.**

Mean reaction times (RT) as a function of task difficulty and accuracy. Task difficulty and accuracy interacted. Error bars represent the standard error of the mean (*N* = 15). ****p* < 0.001


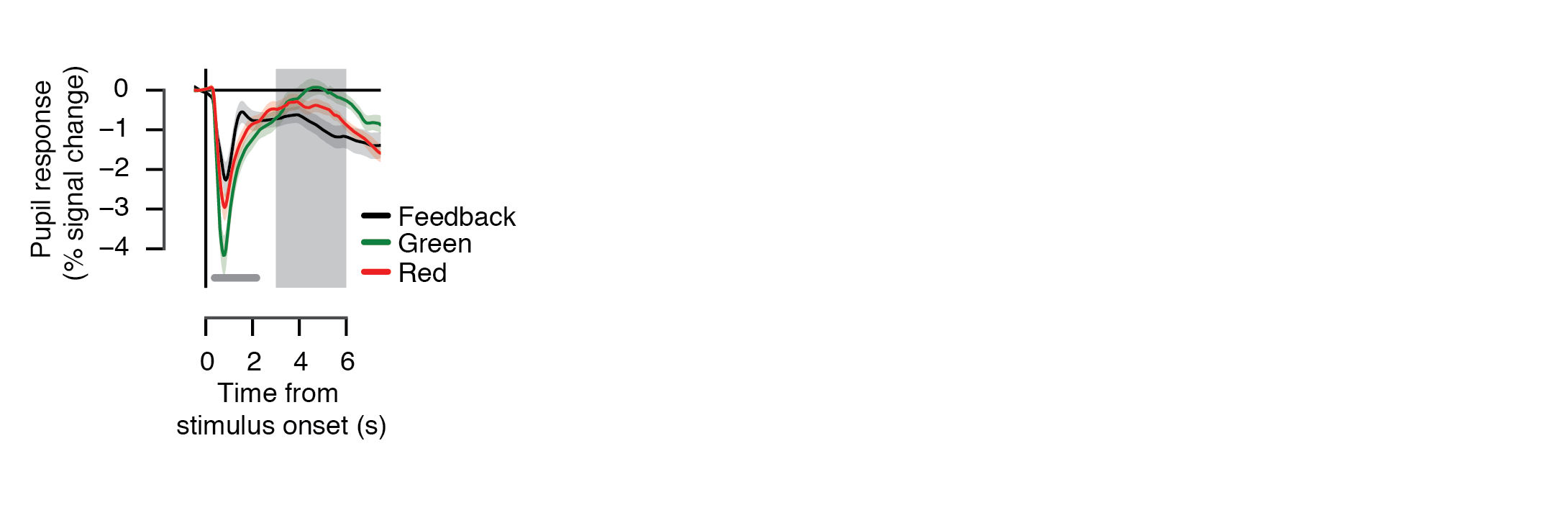


**Supplementary Figure S2. Pupil responses during passive viewing of feedback signals.** In a control experiment (*N* = 15, 5 women, aged 28.5±4 years, range 23-34), we investigated the time course of potential differences in pupil responses evoked by red as compared with green light, regardless of whether these colors correspond to reward feedback during the perceptual choice task. Three subjects were authors, two of which participated in the main experiment. Stimuli were identical to the main 2AFC task; dot motion consisted of only random motion (0% coherence). A trial consisted of a baseline period preceding the onset of a color change (1-3 s, uniformly distributed), the red or green rectangle at fixation (50-50% of trials, randomized, 0.42 s), and ITI (3-6 s, uniformly distributed). Participants passively viewed the stimuli while maintaining fixation. Pupil responses were averaged for each condition of interest per subject (*N* = 15, 200 trials per subject). The light-mediated pupil constrictions evoked by visual feedback cues during the main task (grey) and in the passive viewing control experiment (red, green). The grey bar indicates a difference between red- and green-evoked responses, *p* < 0.05 (cluster-based permutation test, see main text). Grey shaded area, ‘sustained’ time window during which pupil dilation was averaged, defined by the period during which the pupil impulse response function returned to baseline and the shortest delay between events (3-6 s). The results show that (i) green and red light both evoked pupil constrictions, and (ii) green light produced slightly larger pupil constriction than red light, in an early time window (0.25-2.25 s). The difference in Correct vs. Error trials in pupil constriction after feedback during the main experiment continues after this early time window (see Figure 3c). Furthermore, any differences obtained *within* Error and Correct conditions after feedback during the main experiment cannot be explained by differences between the color-evoked responses, as the stimulus color was the same between these comparisons.

**
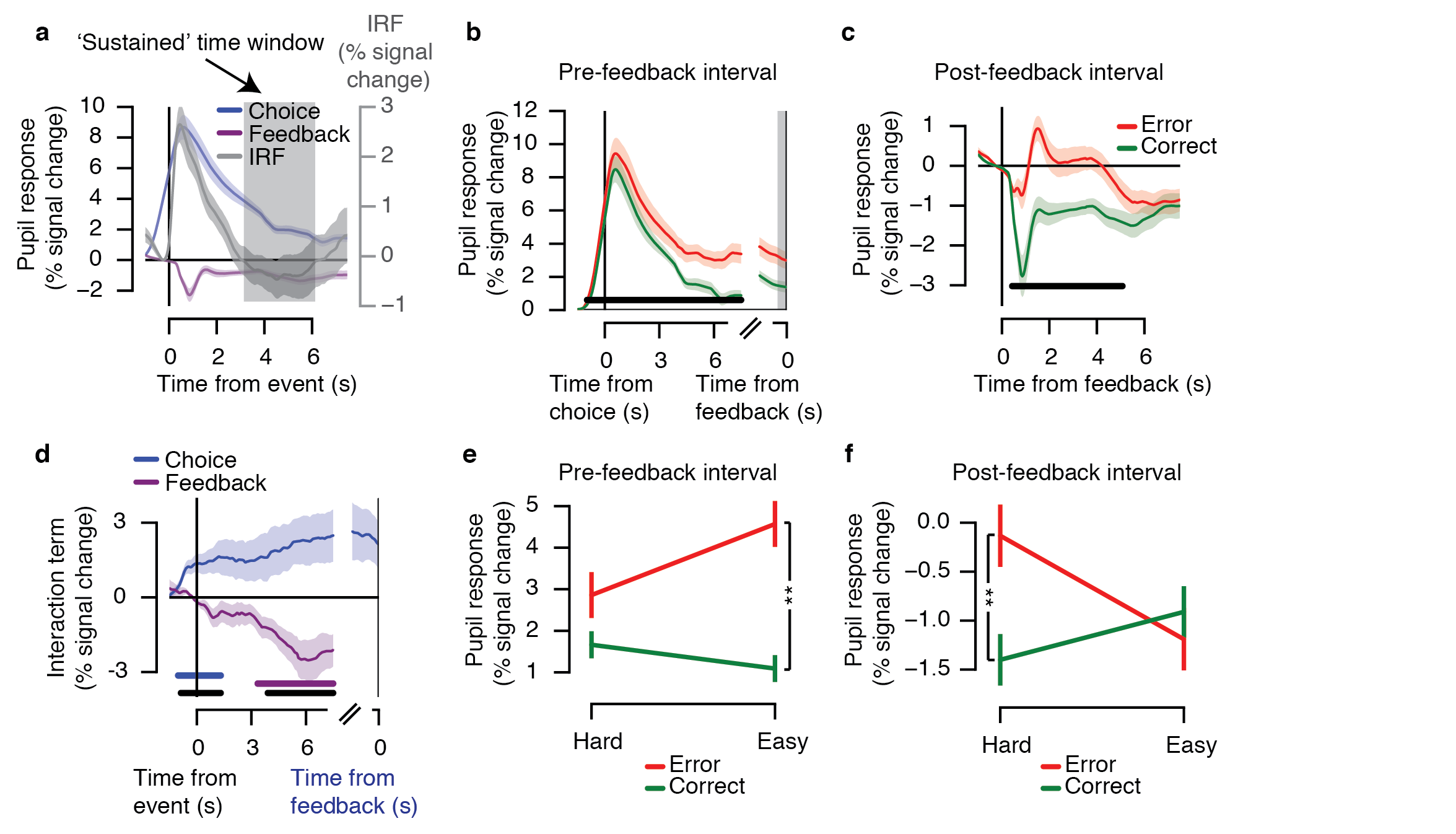
Supplementary Figure S3. Replication of Figure 3 with all trials.** The same pattern of pupil responses was obtained when all trials were included in the analysis, including those in which the interval between events (Delay and ITI periods of a trial; see Fig. 1) was less than 7.5 s (delays ranged from 3.5-11.5 s, with 5 levels in steps of 2 s, uniformly distributed) **(a-f)**. For the pupil responses in the -0.5 s window preceding feedback **(e)**, a significant interaction between difficulty and accuracy was obtained in this later time window (*F*_(1,14)_ = 4.95, *p* = 0.043; post hoc comparisons: Hard Error vs. Hard Correct, *p* = 0.072; Easy Error vs. Easy Correct, *p* = 0.004; Hard Error vs. Easy Error, *p* = 0.074; Hard Correct vs. Easy Correct, *p* = 0.061). During the post-feedback interval, a significant interaction between difficulty and accuracy was obtained **(f)**, *F*_(1,14)_ = 7.89, *p* = 0.014; post hoc comparisons: Hard Error vs. Hard Correct, *p* = 0.001; Easy Error vs. Easy Correct, *p* = 0.572; Hard Error vs. Easy Error, *p* = 0.066; Hard Correct vs. Easy Correct, *p* = 0.118).

**
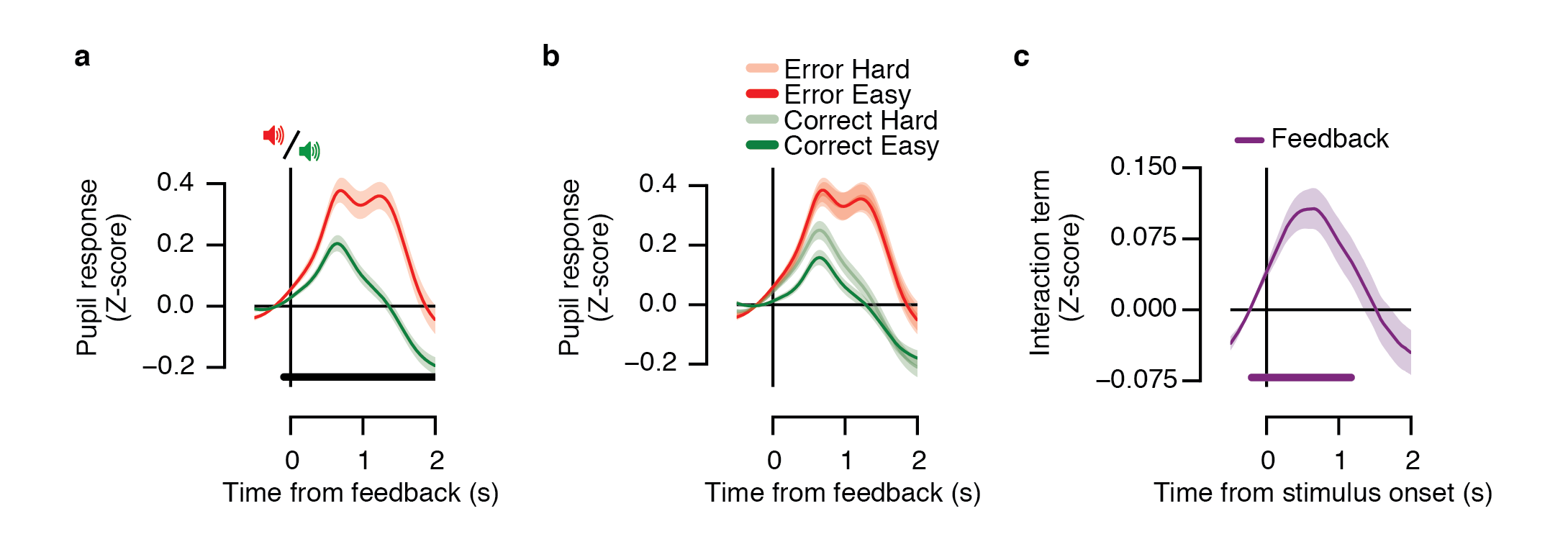
**

**Supplementary Figure S4. Feedback-locked responses from Urai et al. (2017).** Re-analysis of the data from our previously published study (available at <https://doi.org/10.6084/m9.figshare.4300043>). This study used a similar visual perceptual choice task, however with a number of important differences specified in the following: The study used a two-interval forced choice motion coherence discrimination task; multiple levels of task difficulty were intermixed, here sorted into two categories (median split) yielding Hard and Easy conditions for comparison with the present data; delay intervals between decision and feedback, and the inter-trial-intervals were shorter than in the current study; feedback (Correct or Error) was presented by two different tones; feedback was not linked to any reward (participants’ financial remuneration was not contingent on performance). **(a)** Evoked pupil responses for Correct (green) and Error (red) trials locked to trial-wise (auditory) feedback. The black bar indicates Correct vs. Error effect, *p* < 0.05 (cluster-based permutation test). Because feedback was not presented visually, there was no post-feedback pupil constriction, but dilation for all trial types. Error feedback elicited stronger dilations than correct feedback, as in the current data (compare with Figure 3c). **(b)** Pupil responses as a function of task difficulty and accuracy locked to feedback. The scaling with evidence strength was similar to pre-feedback decision uncertainty, but not to post-feedback prediction error, with smaller dilations for Correct Easy than Correct Hard responses (compare to Figure 2c). **(c)** The interaction term for task difficulty with two levels (Easy Error - Easy Correct) - (Hard Error - Hard Correct) for feedback-locked responses. The purple bar indicates the feedback-locked response tested against 0, *p* < 0.05 (cluster-based permutation test). For all feedback-locked responses, the mean pupil diameter across the pre-feedback interval from -0.5 s to 0 s was subtracted from the response time courses at the single-trial level. Each condition of interest was averaged across subjects (*N* = 27).

**
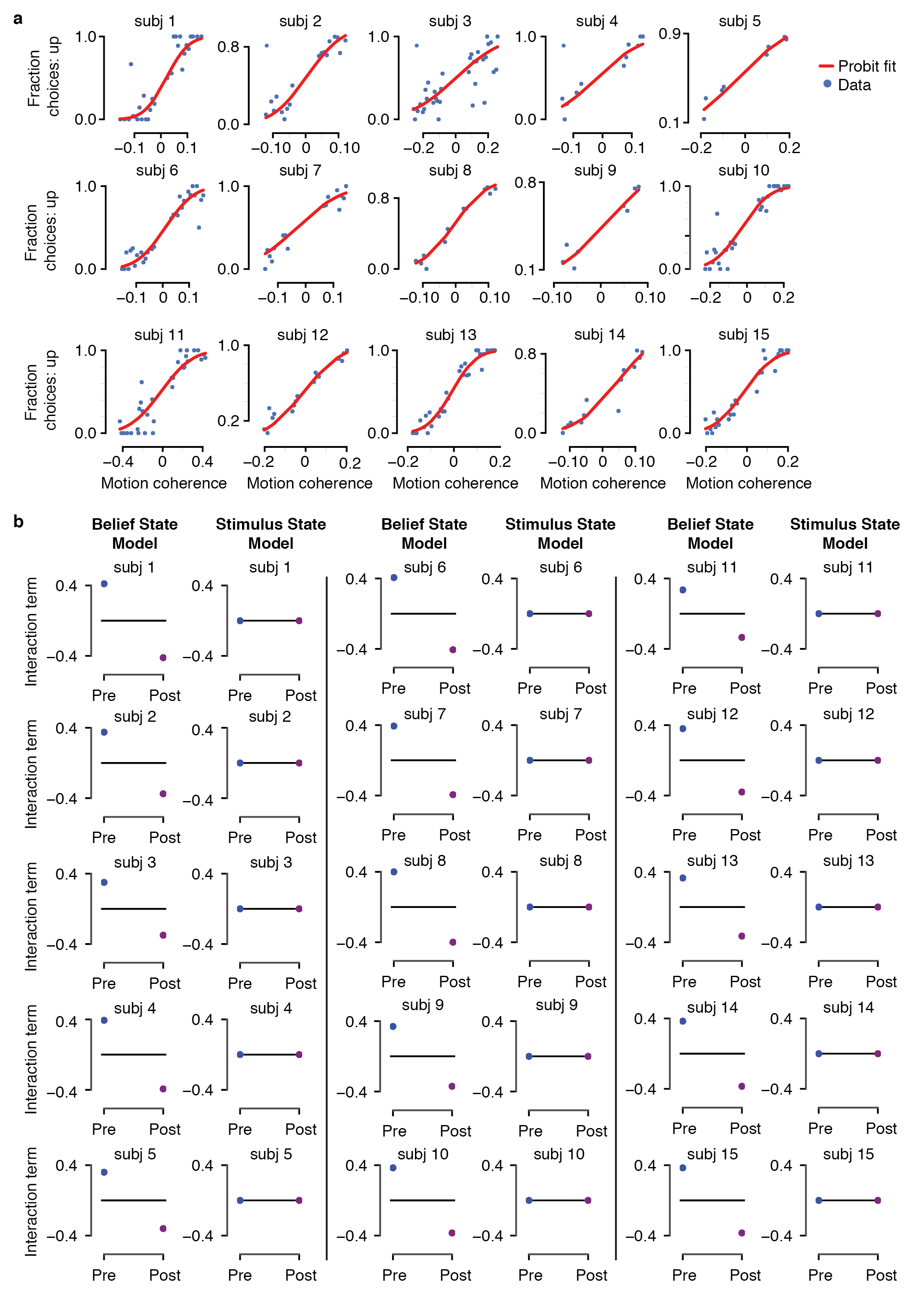
Supplementary Figure S5. Individual psychometric functions and model predictions.** Based on the individual estimates of internal noise in the data (i.e. sigma) **(a)**, subject-specific model predictions were generated for the Belief State and Stimulus State models **(b)**. Predictions for the interaction term defined as (Easy Error - Easy Correct) - (Hard Error - Hard Correct) based on subject-specific motion coherence levels are shown.
